# Protein Kinase B and Extracellular Signal-Regulated Kinase Inactivation is Associated with Regorafenib-Induced Inhibition of Osteosarcoma Progression In Vitro and In Vivo

**DOI:** 10.3390/jcm8060900

**Published:** 2019-06-24

**Authors:** Po-Jung Pan, Yu-Chang Liu, Fei-Ting Hsu

**Affiliations:** 1 Department of Physical Medicine and Rehabilitation, National Yang-Ming University Hospital, Yilan 260, Taiwan; 12105@ymuh.ym.edu.tw; 2 Department of Medicine, National Yang-Ming University, Taipei 112, Taiwan; 3 Department of Radiation Oncology, Chang Bing Show Chwan Memorial Hospital, Changhua 505, Taiwan; 4 Department of Radiation Oncology, Show Chwan Memorial Hospital, Changhua 500, Taiwan; 5 Department of Medical Imaging and Radiological Sciences, Central Taiwan University of Science and Technology, Taichung 406, Taiwan; 6 Department of Biological Science and Technology, China Medical University, Taichung 404, Taiwan

**Keywords:** regorafenib, osteosarcoma, AKT, ERK

## Abstract

Osteosarcoma is the most common type of bone cancer. Multimodality treatment involving chemotherapy, radiotherapy and surgery is not effective enough to control osteosarcoma. Regorafenib, the oral multi-kinase inhibitor, has been shown to have positive efficacy on disease progression delay in chemotherapy resistant osteosarcoma patients. However anti-cancer effect and mechanism of regorafenib in osteosarcoma is ambiguous. Thus, the aim of this study is to investigate the efficacy and molecular mechanism of regorafenib on osteosarcoma in vitro and in vivo. Human osteosarcomas U-2 OS or MG-63 were treated with regorafenib, miltefosine (protein kinase B (AKT) inhibitor), or PD98059 (mitogen-activated protein/extracellular signal-regulated kinase (MEK) pathway inhibitor) for 24 or 48 h. Cell viability, apoptotic signaling transduction, tumor invasion, expression of tumor progression-associated proteins and tumor growth after regorafenib treatment were assayed by MTT 3-(4,5-dimethylthiazol-2-yl)-2,5-diphenyltetrazolium bromide, flow cytometry, transwell assay, Western blotting assay and in vivo animal experiment, respectively. In these studies, we also indicated that regorafenib suppressed cell growth by prompting apoptosis of osteosarcoma cells, which is mediated through inactivation of ERK and AKT signaling pathways. After regorafenib treatment, downregulation of related genes in invasion (vascular endothelial growth factor (VEGF) and matrix metallopeptidase 9 (MMP-9)), proliferation (CyclinD1) and anti-apoptosis (X-linked inhibitor of apoptosis protein (XIAP), myeloid cell leukemia-1 (MCL-1), and cellular FLICE (FADD-like IL-1β-converting enzyme)-inhibitory protein (C-FLIP)) were found. Moreover, upregulation of caspase-3 and caspase-8 cleavage were also observed. In sum, we suggest that regorafenib has potential to suppress osteosarcoma progression via inactivation of AKT and ERK mediated signaling pathway.

## 1. Introduction

Osteosarcoma is the most common type of malignant bone tumor diagnosed in children, adolescents, and adults over 50 years old [1]. The overall survival of patients with localized osteosarcoma has been prolonged with current treatment strategies such as surgery, multi-agent chemotherapy, and radiotherapy. Rapid progression of metastatic osteosarcoma limits efficacy of treatments and correlates with poor prognosis [2,3]. Therefore, development of complementary agents which reduced tumor progression may provide therapeutic benefits for patients with metastatic osteosarcoma.

Hyperactivation of several intracellular signaling pathways promotes tumor progression in osteosarcoma. A number of critical receptor tyrosine kinases, ligands, and intracellular signaling molecules are recognized as targets for therapy in metastatic osteosarcoma [1,4,5]. Sorafenib is the oral multi-kinase inhibitor that targets Raf, mast/stem cell growth factor receptor (c-KIT), vascular endothelial growth factor (VEGF), and platelet-derived growth factor (PDGF) receptor tyrosine kinase signaling transduction and is used for the treatment of hepatocellular carcinoma (HCC), renal cell carcinoma, and differentiated thyroid cancer [6,7]. Efficacy and response of sorafenib in relapsed and unresectable high-grade osteosarcoma after failure of standard multimodal therapy have been demonstrated. The promising results of sorafenib were presented as 4 month progression-free survival, 7 month overall survival, 14% overall response rate, and 49% disease control rate [8].

Regorafenib, the novel sorafenib analogue, is an orally available multi-kinase inhibitor and is approved for the treatment of metastatic colorectal cancer, advanced gastrointestinal stromal tumors, and HCC [9,10]. Efficacy and safety of regorafenib in adult patients with metastatic osteosarcoma have been reported. Regorafenib has presented positive efficacy on delay of disease progression in adult patients with recurrent and progressive metastatic osteosarcoma after failure of conventional chemotherapy [11]. Pignochino et al. found that the inhibition of extracellular signal-regulated kinase (ERK), myeloid cell leukemia-1 (MCL-1), and ezrin pathways contribute to sorafenib blocking osteosarcoma progression in vitro and in vivo [12]. However, the anti-cancer effect and the mechanism of regorafenib in osteosarcoma is ambiguous. The purpose of this present study is to investigate the anti-cancer effect and the mechanism of regorafenib on tumor progression in osteosarcoma in vitro and in vivo.

## 2. Materials and Methods

### 2.1. Chemicals

Regorafenib was provided by Bayer Health Care Pharmaceuticals (Whippany, NY, USA), dissolved in dimethyl sulfoxide (DMSO) (Sigma Chemical Co., St. Louis, MO, USA) as 10 mM stock and stored at −20 °C. AKT inhibitor (Miltefosine) and mitogen-activated protein/extracellular signal-regulated kinase (MEK/ERK) pathway inhibitor (PD98059) were obtained from Selleckchem (Houston, TX, USA).

### 2.2. Cell Culture

Human osteosarcoma U-2 OS and MG-63 cell line were obtained from professor Jing-Gung Chung and Chun-Hsu Yao, respectively, (China Medical University, Taiwan) and maintained at 37 °C under a humidified 5% CO2 atmosphere. Culture medium of U-2 OS cells was 90% McCoy’s 5a medium and contained 2 mM L-glutamine, 10% fetal bovine serum (FBS) and 1% penicillin-streptomycin (PS, 100 U/ml penicillin and 100 µg/mL streptomycin) (Life Technologies, Carlsbad, CA, USA) [13]. Culture medium of MG-63 cells was RPMI-1640 with 10% FBS and 1% PS.

### 2.3. Establishment of Stable U-2 OS/luc2 Cells

JetPEI™ (polyplus-transfection, Illkirch, France) transfection reagent was used to perform pGL4.50 luciferase reporter (pGL4.50 (luc2/CMV)) plasmid transfection on U-2 OS cells. To prepare 500 µL DNA/JetPEI mixture, DNA solution (10 µg pGL4.50 plasmid dissolved in 250 µL of 150 mM NaCl) was mixed with 250 µL JetPEI solution (20 µL of JetPEI reagent diluted in 230 µL of 150 mM NaCl), and then incubated for 30 min. DNA/JetPEI mixture was then added into a 10 cm diameter dish with 80% confluency of U-2 OS cells for 24 h. Then, cells were treated with 200 µg/mL of hygromycin B for 14 days. Viable clones were subcultured into 96-well plates and incubated in cell medium supplemented with 100 µg/mL of hygromycin B. U-2 OS with luc2 function was assayed by IVIS 200 imaging system (Xenogen, Alameda, CA, USA) and named as U-2 OS/*luc2* cells.

### 2.4. 3-(4,5-dimethylthiazol-2-yl)-2,5-diphenyltetrazolium bromide MTT Assay

U-2 OS and MG-63 cells were plated at a density of 3 × 10^4^ cells in a 96-well plate for 24 h and after the 80% confluence was reached, cells were treated with regorafenib at the final concentrations (0, 5, 10, 15, 20 µM) or 0.1% DMSO was used as a vehicle for 24 and 48 h. Cells were stained with MTT (0.5 mg/mL) and then the percentages of viability were readily quantified by absorbance value (OD) at 570 nm. The MTT agent was also purchased from Sigma Chemical Co. [6].

### 2.5. Cell Cycle Analysis

U-2 OS cells (seeded in a 6-well-plate with 5 × 10^5^ cells/well overnight) were treated with 0, 5, 10 µM of regorafenib, 10 µM PD98059, or 10 µM miltefosine for 48 h, respectively. Cells were harvested by trypsinization, centrifugation, fixed in 70% ethanol at 4 °C and then resuspended in propidium iodide staining solution containing 40 µg/mL propidium iodide and 100 µg/mL RNase in PBS at 37 °C for 15 min before analysis by a flow cytometry. Flow cytometric analysis was performed with a flow cytometer (BD Biosciences, FACS Calibur, San Jose, CA, USA) with an excitation at 488 nm and an emission at 630 nm. Five repeated samples were all analyzed by FlowJo software (version 7.6.1; FlowJo LLC, Ashland, OR, USA) [14].

### 2.6. Annexin-V/PI Double Staining for Flow Cytometry Assay

U-2 OS cells (seeded in a 6-well-plate with 5 × 10^5^ cells/well overnight) were treated with 0, 5, 10 µM of regorafenib for 48 h, respectively. Annexin V-FITC apoptosis detection kit was purchased from Vazyme Biotech Co. Lt (Nanjing, China). Next, 100 µL of the cells and binding buffer mixture solution were transferred to a 15 mL culture tube, and incubated with 5 µL of FITC-conjugated annexin-V and 5 µL of PI for 15 min at room temperature in the dark. Finally, a sample was added to each flow cytometry (FACS) tube and analyzed using a flow cytometer and FlowJo software [15].

### 2.7. Caspase-3 and Caspase-8 Activation Analysis

Activity of caspases was measured by flow cytometry using commercially available fluorescent caspase substrates, CaspGlow fluorescein active caspase-3, caspase-8 staining kits (BioVision, Milpitas, CA, USA). Initially, U-2 OS cells (seeded in a 6-well-plate with 5 × 10^5^ cells/well overnight) were treated with 0.1% DMSO (control), 5 µM or 10 µM of regorafenib for 48 h. Cells were harvested, centrifuged and then resuspended in 300 µL of 1 µL substrate solution fluorescein isothiocyanate-Asp(OCH3)-Glu(OCH3)-Val-Asp(OCH3)-fluoromethyl ketone (caspase-3 FITC-DEVD-FMK) or sulforhodamine-Ile-Glu-Thr-Asp-fluoromethyl ketone (caspase-8 Red-IETDFMK) and were incubated at 37 °C under a humidified 5% CO_2_ atmosphere for 60 min. Cells were assayed by caspase-3 and caspase-8 stains as described previously [14,16,17].

### 2.8. Cleavage Poly (ADP-ribose) Polymerase 1 (PARP-1) Activation Analysis

U-2 OS cells were seeded in a 6-well-plate with 5 × 10^5^ cells overnight and treated with 0, 5, 10 µM of regorafenib treatment for 48 h. Cells were washed in PBS, resuspended in 4% formaldehyde fixation buffer for 15 min and refreshed with 90% ice-cold methanol permeabilization buffer overnight at −20 °C. Next, the cells were washed with PBS to remove methanol and resuspended in 100 µL antibody conjugate buffer (2 µL PARP antibody added into 98 µL 0.5% bovine serum albumin buffer) for 15 min [18]. Percentage of cleavage PARP-1 was detected by flow cytometry and five repeated results were analyzed by FlowJo software.

### 2.9. Mitochondria Membrane Potential Analysis

U-2 OS cells (seeded in a 6-well-plate with 5 × 10^5^ cells/well overnight) were treated with 0, 5, 10 µM of regorafenib for 48 h, respectively. Cells were harvested, centrifuged, and resuspended in 500 µL of 3,3′-dihexyloxacarbocyanine iodide (DiOC_6_, 4 µM) for 30 min under 37 °C, for the measurement of mitochondria potential, using flow cytometry as previously described [14].

### 2.10. FAS/FASL Double Staining for Flow Cytometry Assay

U-2 OS cells (seeded in a 6-well-plate with 5 × 10^5^ cells/well overnight) were treated with 0, 5, 10 µM of regorafenib for 48 h, respectively. In brief, cells were harvested, centrifuged, resuspended in binding buffer following having been washed twice with PBS. Then the cells were incubated in FAS-FITC and FASL-PE at room temperature for 15 min. Anti-FAS (FITC) and FAS ligand (PE) flow cytometry antibodies were bought from Thermo Fisher Scientific (Waltham, MA, USA) [19].

### 2.11. Invasion Assay

Transwell (BD Biosciences, Franklin Lakes, NJ, USA) cell culture chambers (8 mm pore size; illipore, Billerica, MA, USA) were uniformly coated with 50 µL BD matrigel matrix on polyethylene terephthalate (PET) membrane and used for cell invasion assay. U-2 OS cells (seeded with 8 × 10^5^ cells/6 cm dish overnight) were treated with 0.1% DMSO (control), 10 µM regorafenib, 10 µM PD98059, or 10 µM miltefosine for 48 h. Cells were then trypsinized, resuspended in a serum-free medium, placed in the upper chamber of the transwell insert and the lower chamber was filled with complete medium (90% McCoy’s 5a medium containing 10% FBS). Cells were allowed to invade at 37 °C for 48 h. The nonmigrated cells were removed and the invaded cells in the PET membrane were fixed with 4% formaldehyde in PBS and were then stained with 0.5% crystal violet. The bottoms of the PET membranes were photographed and quantified under a light microscope at ×100 in five random fields per membrane [14,20].

### 2.12. In Vitro and Ex Vivo Western Blot

U-2 OS and MG-63 cells (plated with 1 × 10^6^ cells/10 cm dish overnight) were treated with 0, 5, 10 µM of regorafenib, 10 µM PD98059, or 10 µM miltefosine for 48 h, respectively. Mice were sacrificed on day 15 after treatment and then tumors were isolated from mice. U-2 OS cells or U-2 OS tumor tissue were lysed by RIPA lysis buffer (Abcam, Cambridge, UK). The experimental procedure of Western blot was followed as described previously [14,20]. In brief, 40–80 µg total proteins were separated by polyacrylamide gel electrophoresis, transferred onto polyvinylidene difluoride (PVDF) membranes (EMD Millipore, Bedford, MA, USA) and were incubated with primary antibodies. The primary antibodies used were: Phospho-p44/42 MAPK (Erk1/2) (P27361, 1:1000, rabbit, Cell signaling, Danvers, MA, USA), tERK (sc-154, 1:1000, rabbit, Santa Cruz, CA, USA), VEGF (ab1316, 1:1000, mouse, Abcam), Phospho-Akt (Ser473) (P31749, 1:1000, rabbit, Cell signaling), Akt1/2/3 (sc-8312, 1:1000, rabbit, Santa Cruz), MMP-9 (AB19016, 1:1000, rabbit, EMD Millipore), XIAP (PA5-29253, 1:1000, rabbit, Thermo Fisher Scientific), CyclinD1 (DCS-6, 1:1000, mouse, Thermo Fisher Scientific), MCL-1 (BV-438, 1:1000, rabbit, BioVision), C-FLIP (D16A8, 1:1000, rabbit, Cell signaling), and β-actin (sc-47778, 1:1000, mouse, Santa Cruz). Membranes were stained with the appropriate secondary antibodies (GeneTex, Irvine, CA, USA) and were visualized with enhanced chemiluminescence detection (Millipore, Temecula, CA, USA). Image J 1.490 software (National Institutes of Health, Bethesda, MD, USA) was used to quantify changes in protein expression by densitometry analysis and using β-actin as the loading control.

### 2.13. Animal Experiment and U-2 OS Xenograft Animal Model

The experimental procedure was described in Figure section. Mouse experiments were conducted under ethics approval from China Medical University Animal Ethics Committee (Permit Number: CMU IACUC-2019-018). Six week old male NOD/SCID mice were purchased from the National Laboratory Animal Center (Taipei, Taiwan). First, 1 × 10^7^ U-2 OS/*luc2* cells (200 µL of 1: 1 mixed cell suspension and matrigel) were subcutaneously injected into the left flanks of mice to establish a U-2 OS xenograft animal model [21]. A total of 20 mice were randomly divided into 2 groups after tumors reached 100 mm^3^, including non-treated control (CTRL, 0.1% DMSO) and regorafenib (10 mg/kg/day by gavage for 14 days). Tumor volumes were measured using a digital caliper, recorded and expressed in mm^3^, using the formula, volume (mm^3^) = (0.523) × length (mm) × width (mm) × height (mm). Mice were sacrificed on day 15 for further experiment.

### 2.14. Animal Bioluminescent Imaging

In vivo bioluminescent imaging (BLI) was performed on day 0 and 14 after regorafenib treatment. A 30-gauge needle was carefully inserted into the peritoneal and injected with D-luciferin (150 mg/kg) 15 min before image acquisition. Then, mice were anesthetized by 1%–3% isoflurane, scanned by Xtreme (Bruker, Billerica, MA, USA) and signal intensities were quantified by using molecular imaging software version 7.2 (Bruker) [22].

### 2.15. Histological Analysis

Mice tumor tissues were fixed in 10% phosphate-buffered formalin for 2 days and dehydrated in a series of graded alcohols (50%, 70%, and 95%) for 30 min each. Samples were then embedded in paraffin, cut into 5 µm sections, and stained with hematoxylin and eosin (H&E). Images were taken using a light a microscope (Nikon ECLIPSE Ti-U) at ×100 [22].

### 2.16. Statistical Analysis

All data are expressed as the mean ± standard deviation (SD) from at least 3 experiments. Differences between groups were analyzed by student’s t-test excel 2017 (Redmond, Washington, United States). *p*-Value <0.05 was considered as statistically significant.

## 3. Results

### 3.1. Regorafenib-Induced Cytotoxicity of U-2 OS Human Osteosarcoma Cells

**Figure 1:**
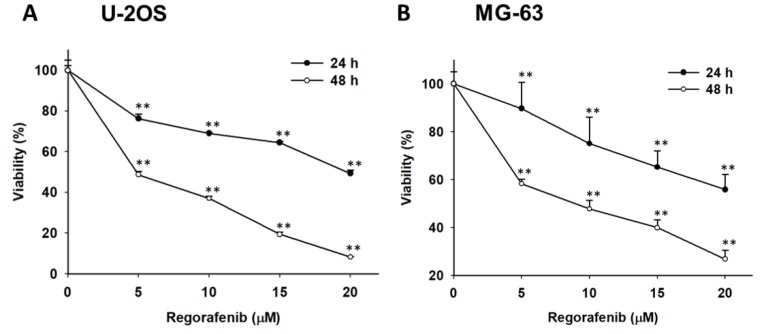
The viability of human osteosarcoma cells was reduced by regorafenib. (**A**) U-2 OS cells and (**B**) MG-63 cells were treated with 0, 5, 10, 15, 20 µM of regorafenib for 24 h or 48 h. The viability of U-2 OS cells and MG-63 cells were both evaluated by 3-(4,5-Dimethylthiazol-2-yl)-2,5-diphenyltetrazolium bromide (MTT) assay. * *p* < 0.05 and ** *p* < 0.01 vs. 0 µM of regorafenib.

To investigate whether regorafenib may be a potential anti-osteosarcoma agent, we treated U-2 OS and MG-63 cells with various concentrations of regorafenib (0–20 µM) for 24 h and 48 h. As showed in Figure 1A,B, cytotoxicity of U-2 OS and MG-63 was increased by time and dose of regorafenib treatment. The inhibitory concentration (IC) IC_50_ and IC_60_ of regorafenib on U-2 OS and and MG-63 cells was between 5 µM and 10 µM after 48 h administration. Here, we suggested that regorafenib may induce cytotoxicity of two types of osteosarcoma cells. Thus, we further investigated treatment efficacy and molecular mechanism of regorafenib on osteosarcoma by using 5 µM and 10 µM for 48 h.

### 3.2. Regorafenib-Induced Apoptosis and DNA Damage of U-2 OS Human Osteosarcoma Cells

**Figure 2:**
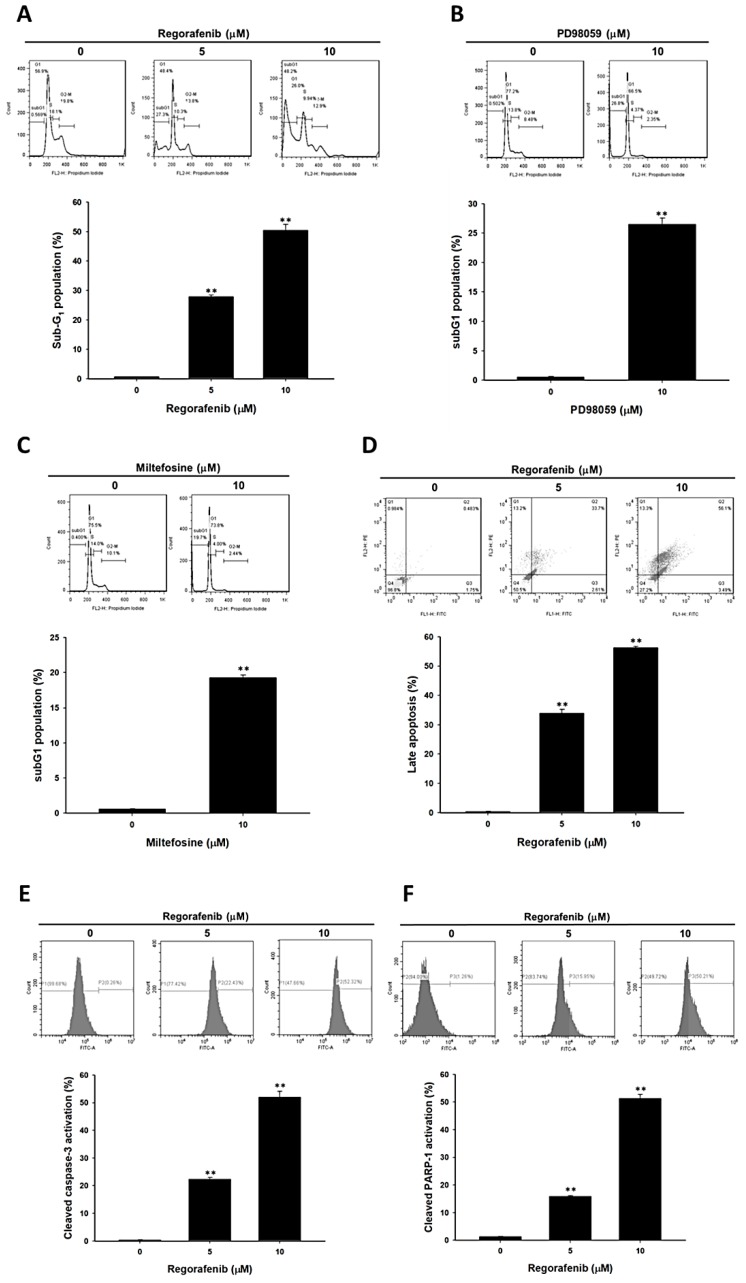
Apoptosis and DNA damage effect were increased by regorafenib. U-2 OS cells was treated with 0, 5, 10 µM of regorafenib, 10 µM of PD98059 or 10 µM of miltefosine for 48 h, respectively. The apoptosis effect was executed by flow cytometry. Cells were respectively stained with (**A**–**C**) PI for cell cycle; (**D**) annexin-V and PI for late apoptosis; (**E**) caspase-3 for apoptosis and; (**F**) cleaved PARP-1 for DNA damage. ** *p* < 0.01 vs. 0 µM of regorafenib.

Next, we evaluated whether regorafenib may be able to mediate the apoptosis effect of U-2 OS cells. Here, we performed along with cell cycle subG1 analysis, annexin-V/PI staining, and caspase-3 activity assay to investigate the apoptosis effect of regorafenib by 5 µM and 10 µM treatment for 48 h. Figure 2 A indicates the accumulation of the subG1 population was increased to 30–50% in the regorafenib treatment group. Annexin-V and PI double positive was recognized as late apoptosis which was also induced by regorafenib (Figure 2D). We also validated the subG1 population change by treatment with various inhibitors, such as 10 µM of PD98059 or 10 µM of miltefosine for 48 h. Thus, we indicated that the inhibition of ERK and AKT may trigger the accumulation of subG1 population, which is represented as apoptosis effect (Figure 2B,C). Furthermore, the cleaved caspase-3 was markedly activated by 5 µM and 10 µM regorafenib treatment (Figure 2E). In addition, a DNA damage marker, cleavage of PARP-1 activity, was significantly greater than the untreated group (Figure 2F). In sum, our results indicated that regorafenib may trigger apoptosis and DNA damage effect of osteosarcoma cells.

### 3.3. Regorafenib-Induced Apoptosis was Dependent on Both Extrinsic and Intrinsic Pathways

**Figure 3:**
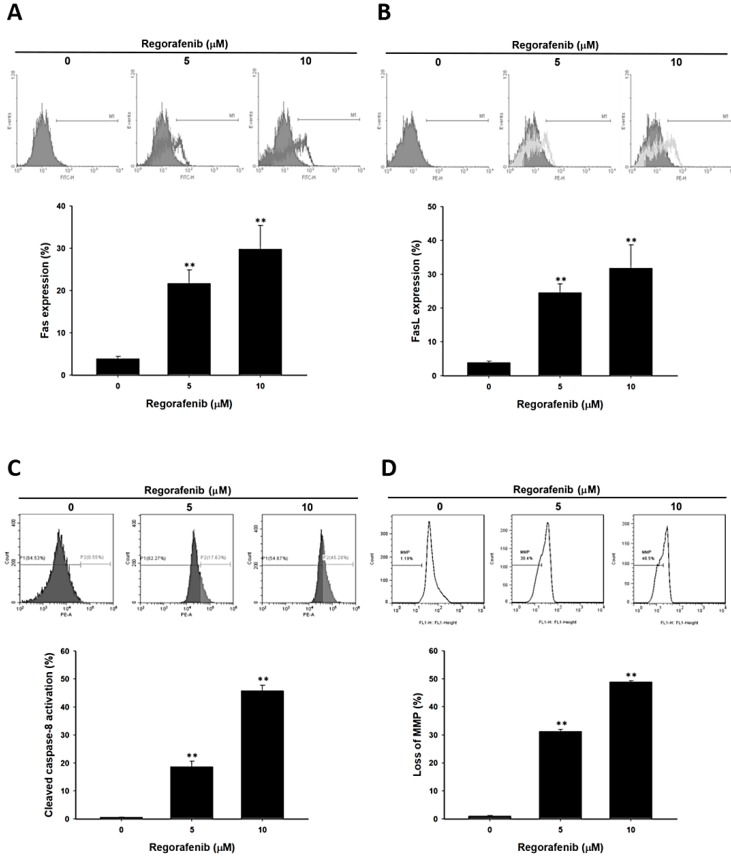
The death receptor dependent and mitochondria dependent apoptosis were activated by regorafenib. U-2 OS cells was treated with 0, 5, 10 µM of regorafenib for 48 h. The activation of (**A**) FAS, (**B**) FASL, and (**C**) cleaved caspase-8 were all detected by flow cytometry. (**D**) Mitochondria membrane potential was stained by DiOC_6_ and also assayed by flow cytometry. ** *p* < 0.01 vs. 0 µM of regorafenib.

In order to understand the mechanism of regorafenib-induced apoptosis, we investigated the change of extrinsic and intrinsic apoptosis makers. Death receptor (FAS) and their ligand FASL were both increased by regorafenib (Figure 3A,B). Moreover, the activation of cleaved caspase-8 was also found in regorafenib treated U-2 OS cells (Figure 3C). The expression of these three markers suggested that regorafenib induced an extrinsic apoptosis signal. Furthermore, we indicated that regorafenib also increased the loss of mitochondria membrane potential which was defined as intrinsic apoptosis signaling (Figure 3C). Taken together, regorafenib triggered death receptor dependent and mitochondria dependent apoptosis effects in human osteosarcoma cells.

### 3.4. Regorafenib Suppressed Tumor Progression via Blocking ERK and AKT Signaling Transduction

**Figure 4:**
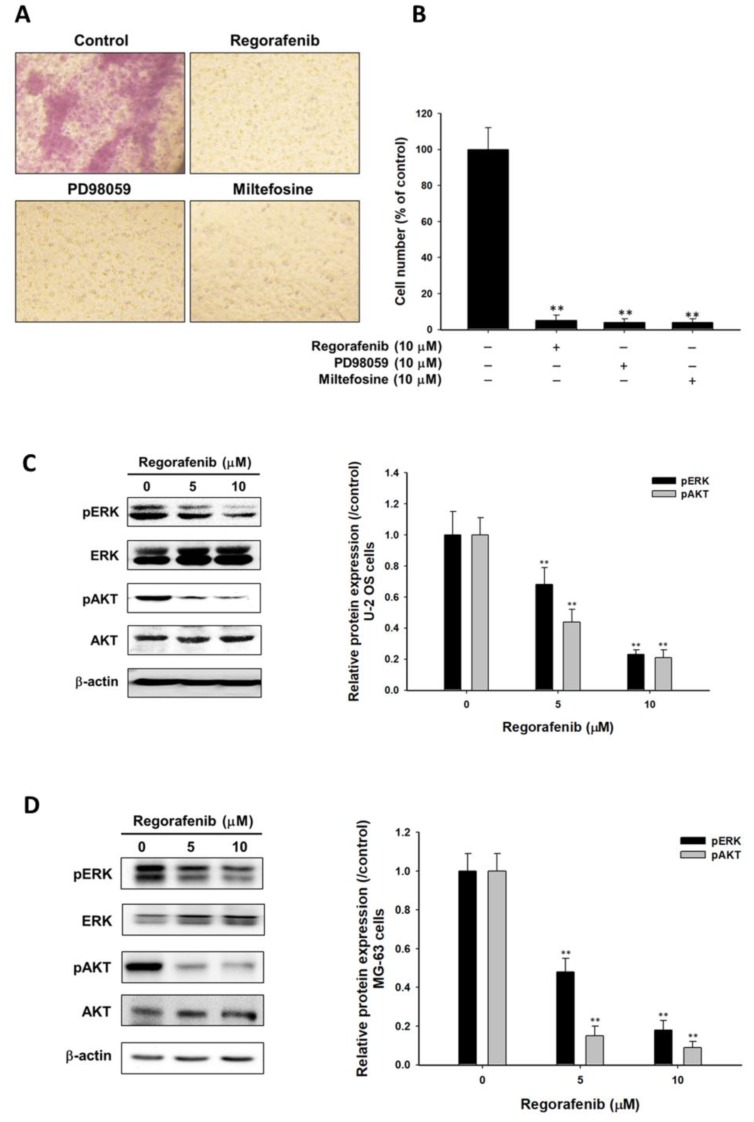

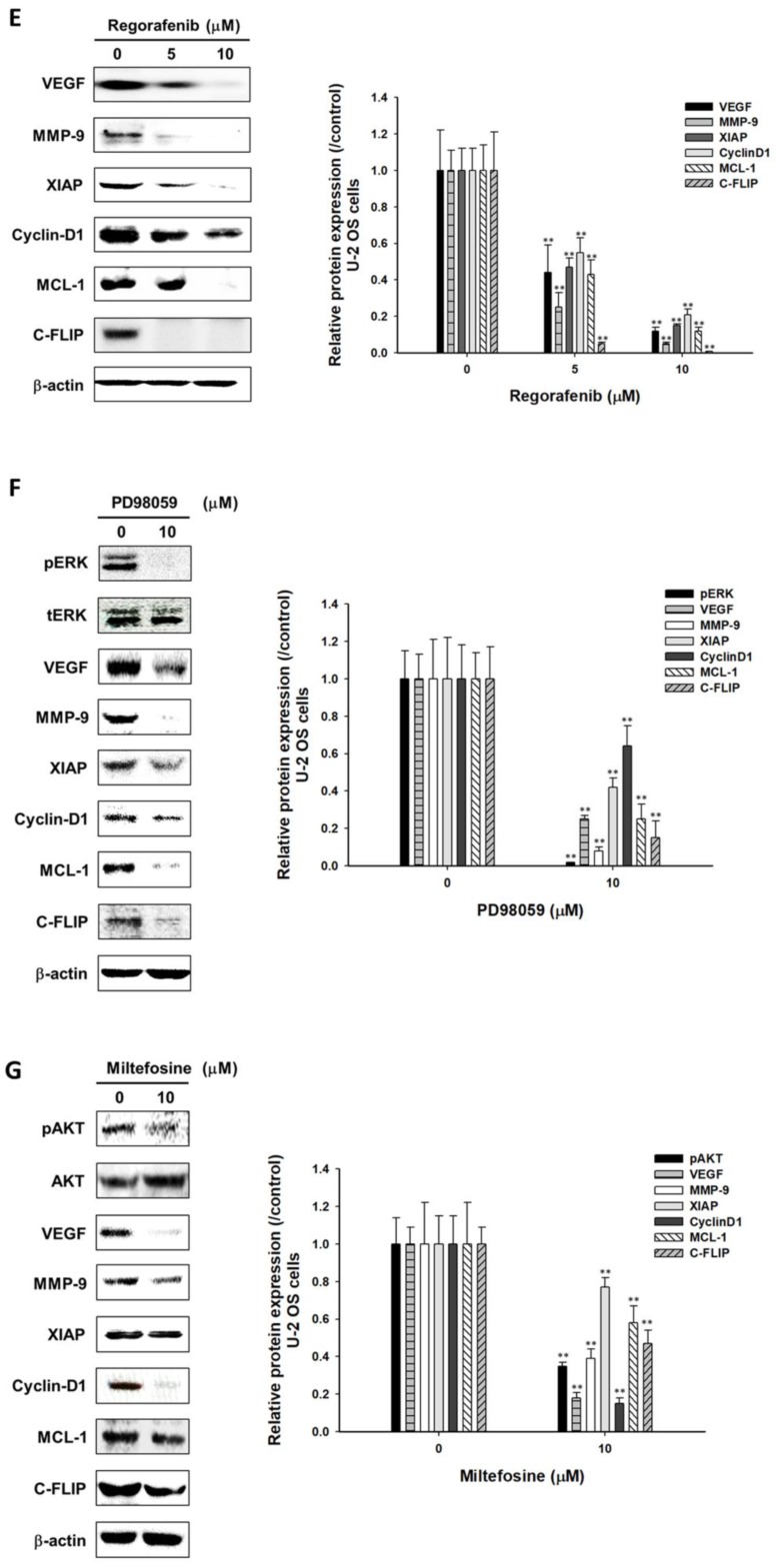
Tumor progression was blocked by regorafenib via inhibition of ERK and AKT signaling. U-2 OS cells were treated with 10 µM of regorafenib, 10 µM of PD98059 or 10 µM of miltefosine for 48 h and followed by invasion assay. MG-63 cells were treated with 10 µM of regorafenib. (**A**,**B**) Light view photographed images and quantification bar chart of each group. Three independent experiments of protein expression level of pERK and pAKT were detected and quantified after 0, 5 or 10 µM of regorafenib treatment in (**C**) U-2 OS cells or (**D**) MG-63 cells. Protein level of VEGF, MMP-9, XIAP, CyclinD1, MCL-1 and C-FLIP of U-2 OS cells were measured after various treatments, including (**E**) 0, 5 or 10 µM of regorafenib; (**F**) 10 µM of PD98059; and (**G**) 10 µM of miltefosine. ** *p* < 0.01 vs. 0 µM treatment group.

First, transwell assays were conducted to assess the invasion ability of U-2 OS cells after regorafenib treatment. Number of invaded U-2 OS cells was reduced by 10 µM regorafenib treatment (Figure 4A,B). Next, PD98059 (MEK/ERK pathway inhibitor) and Miltefosine (AKT inhibitor) were both used as positive controls to prove that the suppression of ERK and AKT signaling may also reduce the invasion ability of U-2 OS cells. Notably, we found that regorafenib markedly suppressed the phosphorylation of ERK and AKT on two types of osteosarcoma cells (Figure 4C,D). Moreover, the protein level of angiogenesis related protein VEGF, metastasis-associated proteins MMP-9, proliferation related proteins (CyclinD1) and anti-apoptosis related proteins (XIAP, MCL-1 and C-FLIP) were all dropped after regorafenib treatment (Figure 4E). The results showed in Figure 4F and G both imply that the blockage of ERK and AKT signaling may facilitate the suppression of tumor progression. We may infer that the dephosphorylation of ERK and AKT by regorafenib finally resulted in the reduction of various tumor progression factors. In sum, osteosarcoma progress was inhibited by regorafenib via inactivation of AKT and ERK pathways.

### 3.5. Regorafenib Markedly Repressed the Growth of U-2 OS Human Osteosarcoma

**Figure 5:**
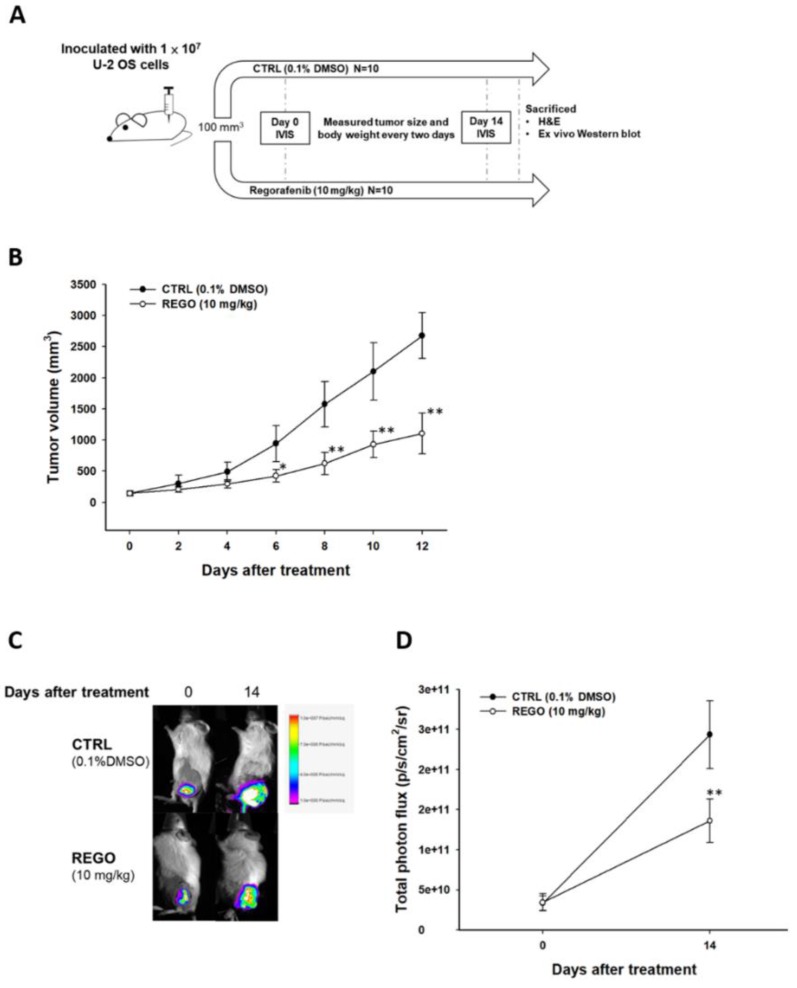

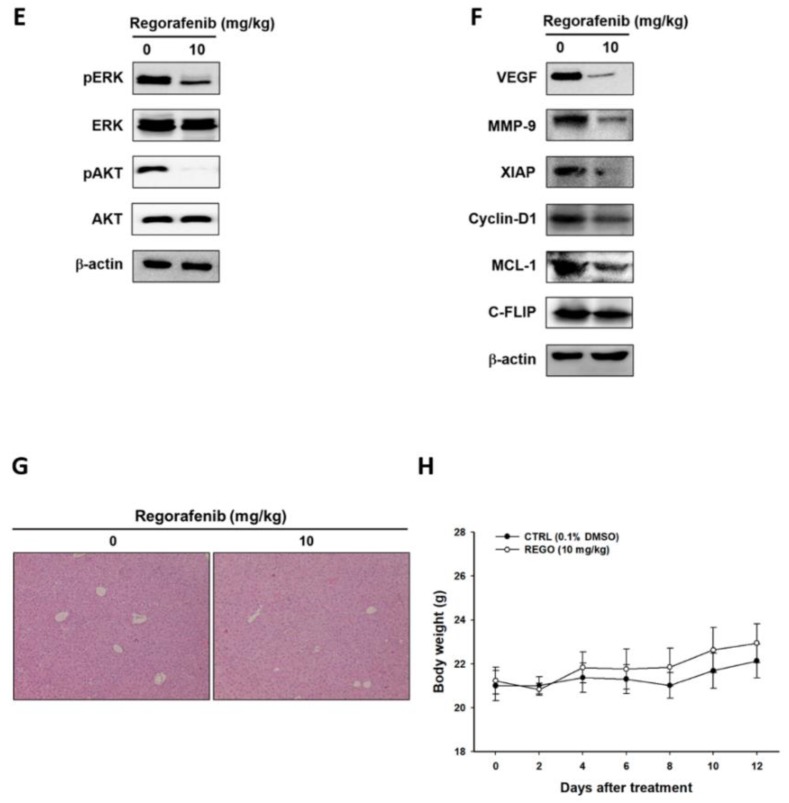
Tumor growth was suppressed by regorafenib treatment. (**A**) Experimental flow chart of animal study. (**B**) Tumor size measured by caliper from day 0 to day 12. (**C**) Bioluminescent imaging (BLI) of each group on day 0 and day 14 after treatment. (**D**) Photon intensity quantification bar chart of each group. (**E**) Ex vivo Western blot result of ERK and AKT. (**F**) Protein level of VEGF, MMP-9, XIAP, CyclinD1, MCL-1 and C-FLIP which were extracted from mice tumor tissue. (**G**) Hematoxylin and eosin (H&E) stain of liver tissue. (**H**) Body weight record of mice of every two days during therapy. * *p* < 0.05 and ** *p* < 0.01 vs. CTRL.

Finally, we evaluated the therapeutic efficacy of regorafenib on a U-2 OS bearing animal model and designed the experiment depicted in Figure 5A. The volume of the tumor was markedly suppressed and controlled by regorafenib 6 days after treatment (Figure 5B). Then, we further investigated the living cell signal from U-2 OS/*luc2* bearing animal model. Figure 5C,D signal that the intensity from viable U-2 OS cells was relatively less in the regorafenib treatment group compared to the non-treatment control. This results corresponds to our tumor volume evaluation. Furthermore, we investigated the alteration of key regulators by regorafenib using ex vivo Western blot. The activation form of pERK protein and pAKT protein levels were all diminished by regorafenib (Figure 5E). Moreover, tumor progression related proteins were all downregulated by regorafenib as showed in Figure 5F. However, there were no changes of liver pathology and mice body weight after regorafenib administration (Figure 5G,H). Taken together, regorafenib may effectively trigger osteosarcoma growth inhibition via blocking ERK and AKT signaling without causing any general toxicity.

## 4. Discussion

ERK is a key member in the Ras/Raf/MEK/ERK signaling pathway and can be activated by the dual specificity of kinase MEK1/2. Both mitogen-activated protein kinases (MAPK)/extracellular signal-regulated kinases (ERK) and AKT/protein kinases B (PKB) signaling promote cell proliferation, survival, angiogenesis, and metastasis through phosphorylating downstream proteins and transcription factors in cancers. High expression of ERK and AKT phosphorylation was found in osteosarcoma and associated with a poor outcome. Suppression of ERK and AKT phosphorylation is conducive to anticancer agents to inhibit tumor progression in osteosarcoma in vitro and in vivo [23,24,25,26]. Everolimus, the inhibitor of the mammalian target of rapamycin (mTOR), has been shown to inhibit tumor cell growth through suppression of phosphoinositide 3-kinase (PI3K)/AKT pathway in breast cancer [27]. The combination of sorafenib and everolimus showed anti-cancer activity as a further-line treatment for patients with unresectable high-grade osteosarcoma progressing after standard treatment [28]. We found the toxicity of osteosarcoma was induced by regorafenib (Figure 1). Our current results also presented that both PD98059 (MEK/ERK pathway inhibitor) and miltefosine (AKT inhibitor) both inhibit the expression of tumor progression-associated proteins (VEGF, MMP-9, Cyclin-D1, C-FLIP, MCL-1, and XIAP) in osteosarcoma U-2 OS cells in vitro (Figure 4F,G). In our previous studies, we indicated that regorafenib inhibited ERK and AKT phosphorylation in HCC in vitro and in vivo [9,21]. In this present study, we also proved that regorafenib reduces protein levels of pERK and pAKT (Ser473) in osteosarcoma U-2 OS cells and MG-63 cells (Figure 4C,D).

Vascular endothelial growth factor (VEGF) is the essential mediator in tumor angiogenesis, growth, and metastasis. High expression of VEGF contributes to tumor growth and correlates with pulmonary metastasis and poor prognosis in patients with osteosarcoma [29]. Matrix metalloproteinase 9 (MMP-9) and gelatinase B, modulates cell growth, invasion, and metastasis leading to tumor progression. Zhou et al. found positive expression of MMP-9 was linked to neoplasm metastasis and poor survival in osteosarcoma [30]. Cyclin-D1 as the transcriptional co-regulator controls cell cycle progression. Cyclin-D1 has been indicated to regulate cell migration/invasion, promote cell proliferation and inhibition of apoptosis in osteosarcoma [31,32]. Many anti-apoptotic proteins such as C-FLIP, MCL-1, and XIAP inhibit apoptosis through disruption of extrinsic/intrinsic apoptotic pathways in cancers [33]. C-FLIP inhibits FAS-mediated apoptosis through suppression of caspase-8 activation [34]. MCL-1 blocks intrinsic apoptotic signaling transduction through preventing the loss of mitochondrial membrane potential and cytochrome-c release from mitochondria [35]. XIAP diminishes anti-cancer agents-induced apoptosis via inhibition of caspase-3 activation [36]. Tumor progression-associated proteins (VEGF, MMP-9, Cyclin-D1, C-FLIP, MCL-1, and XIAP) may serve as therapeutic targets for inhibition of osteosarcoma progression. Our results demonstrated that regorafenib may effectively suppress the expression of tumor progression-associated proteins (Figure 4E).

Inhibition of tumor progression-associated proteins by small interfering or micro RNA (siRNA or miRNA) reduces cell growth, angiogenesis, and invasion and enhances anti-cancer agents-induced apoptosis in osteosarcoma [37,38,39,40]. Pignochino et al. indicated sorafenib induces cytotoxicity and apoptosis and reduces protein expression of pERK and MCL-1, and inhibits protein secretion of VEGF and MMP-2 in osteosarcoma [12]. In Figure 2A and D–F, we proved that regorafenib, an analogue of sorafenib may also induce apoptosis effects of osteosarcoma. In addition to inhibition of ERK and AKT phosphorylation, we also found regorafenib reduced tumor invasion capacity. As showed in Figure 4A, our results indicated that the invasion ability was markedly reduced by regorafenib, AKT and MEK/ERK pathway inhibitors. Knockdown of ERK and AKT by siRNA induces apoptosis and sensitizes osteosarcoma cells to cisplatin [35,36]. We found both PD98059 and miltefosine-induced apoptosis in osteosarcoma U-2 OS cells (Figure 2B, C). In addition, our results also implicated that both extrinsic and intrinsic apoptotic signaling transduction may act critically in regorafenib-induced apoptosis in osteosarcoma (Figure 3). The results from phase 2 studies in adult patients with metastatic osteosarcoma presented that regorafenib improves progression-free survival (62% at 12 weeks and 35% at 24 weeks) and suggested regorafenib has an important therapeutic role as a complementary agent supporting standard cytotoxic chemotherapy in the treatment of osteosarcoma [11].

## 5. Conclusions

In conclusion, ERK and AKT dephosphorylation are linked to regorafenib-induced apoptosis, the reduction of tumor growth and invasion, and the suppression of tumor progression-associated proteins. We suggested that ERK inactivation and AKT inactivation are critical factors for regorafenib-induced inhibition of osteosarcoma progression in vitro and in vivo.
